# Population Genetic Structure of *Anisakis simplex* Infecting the European Hake from North East Atlantic Fishing Grounds

**DOI:** 10.3390/ani13020197

**Published:** 2023-01-04

**Authors:** Andrea Ramilo, Helena Rodríguez, Santiago Pascual, Ángel F. González, Elvira Abollo

**Affiliations:** Instituto de Investigaciones Marinas, Consejo Superior de Investigaciones Científicas, IIM-CSIC, 36208 Vigo, Pontevedra, Spain

**Keywords:** *Anisakis simplex*, *Anisakis pegreffii*, hybrid genotype, European hake, genetic structure

## Abstract

**Simple Summary:**

The nematodes of the genus *Anisakis* are among the most prevalent parasites found in fishes and marine mammals and they are the main cause of human anisakiasis. Genetic studies have described two species, *A. simplex* and *A. pegreffii*, in European waters parasitizing several hosts, being the European hake, by far, the fish with higher infection values. The aim of this study is to enhance the knowledge of the distribution and population structure of the *Anisakis* species infecting hakes from the major European fishing areas through the use of genetic analysis. This study provides useful information about the genetic diversity of these parasites in the different fishing areas and fish tissues, valuable findings to understand the parasite speciation to different hake tissues and how they are structured along European waters.

**Abstract:**

The European hake, one of the most commercially valuable species in ICES fishing areas, is considered an important neglected source of zoonotic risk by nematode parasites belonging to the genus *Anisakis*. *Merluccius merluccius* is, by far, the most important host of *Anisakis* spp. at the European fishing grounds, in terms of demographic infection values, and carries the highest parasite burden. These high parasite population densities within an individual fish host offer a chance to explore new sources of variations for the genetic structure of *Anisakis* spp. populations. A total of 873 *Anisakis* spp. third-stage larvae, originally sampled from viscera and muscular sections of hake collected at ten fishing grounds, were primarily identified using ITS rDNA region as molecular marker. After that, we used mtDNA *cox2* gene to reveal the high haplotype diversity and the lack of genetic structure for *A. simplex*. Dominant haplotypes were shared among the different fishing areas and fish sections analyzed. Results indicate a clear connection of *A. simplex* from European hake along the Northern North Sea to the Portuguese coast, constituting a single genetic population but revealing a certain level of genetic sub-structuring on the Northwest coast of Scotland. This study also provides useful information to advance the understanding of parasite speciation to different fish host tissues or microenvironments.

## 1. Introduction

The European hake *Merluccius merluccius* (Linnaeus, 1758) (Gadiformes, Merlucciidae) is one of the most commercially important species in the North East Atlantic fishing area (FAO27). Nowadays, the International Council for Exploration of the Sea (ICES) considers two different stocks in EU Atlantic waters separated by the Capbreton Canyon. The northern hake stock comprises the North Sea, Skagerrak and Kattegat, the coast of UK, France and Ireland, while the southern stock is distributed along the Atlantic coast of Spain and Portugal [1,2]. This demersal predator is largely overexploited by industrial fisheries since it is a product much prized by European markets, supplied either whole or filleted. At the same time, the European hake tops the list of fish species with highest exposure risk by zoonotic parasites of the genus *Anisakis* Dujardin, 1845 (Rhabditida, Anisakidae) for seafood consumers [3,4,5]. Furthermore, this noticeable high abundance of *Anisakis* in hake fillets results in the rejection of parasitized fish products by official inspectors in the industry. Moreover, the presence of *Anisakis* spp. in fish edible parts gives rise to the reduction of the marketability of aesthetically unattractive fish, with significant economic losses for European hake markets [3,4,6]. 

The genetic population structure studies of parasites have contributed to clarify the taxonomy of cryptic species, morphologically indistinguishable, which is common in nematodes of the genus *Anisakis*. To date, nine species of this genus have been identified, using different diagnostic genetic markers, showing that they can be grouped in four distinct clades. Two species of clade 1, *A. simplex* (s. s.) (Rudolphi, 1809) Dujardin, 1845 and *A. pegreffii* Campana-Rouget & Bioca, 1955, have been reported up to now in several European fishing areas and in several intermediate and definitive hosts [3,7,8,9,10,11,12,13]. *A. simplex* is the most prevalent species in FAO27 and coexists with *A. pegreffii* in Spanish and Portuguese coasts, even hybrids between them have been found in these sympatric areas [8,14,15]. 

The recruitment and accumulation of *Anisakis* spp. larvae in European hakes is enhanced by their key trophic role in NE Atlantic ecosystems. Hakes feed on mesozooplankton (euphausiids and amphipods), small zooplanctivorous fish and larger demersal prey, which are all well known as intermediate and paratenic hosts of *Anisakis* spp. [12,16]. Likewise, hake is an important prey for the definitive host of the parasite, particularly dolphins, thus reinforcing the life cycle of *Anisakis* spp. [7,11]. Therefore, the genetic population structure of *Anisakis* spp. in any particular fish species can be affected by changes in the food-web structure, including the role of anthropogenetic impacts on the exploited fish stocks and ecosystems. 

Beyond the impact on human health and on food industry, the study of the genetic population structure of parasites also provides information about their dynamics of infection as their host-specificity, their speciation to different tissues in a single host or their ability to adapt to local environments and to climate change [17,18,19]. It has also been described that the genetic structure of parasites could give information about the population genetic structure of their hosts, and thus, they could be used as biological tags for stock identification of fish species [20].

This study has a double-aim: (1) to genetically identify the *Anisakis* spp. larvae collected from European hake from the most significant fishing grounds of ICES areas and (2) to establish the genetic diversity and the population genetic structure of *A. simplex* in relation to fish origin and site of infection.

## 2. Materials and Methods

### 2.1. Fish and Parasite sampling

**Figure 1:**
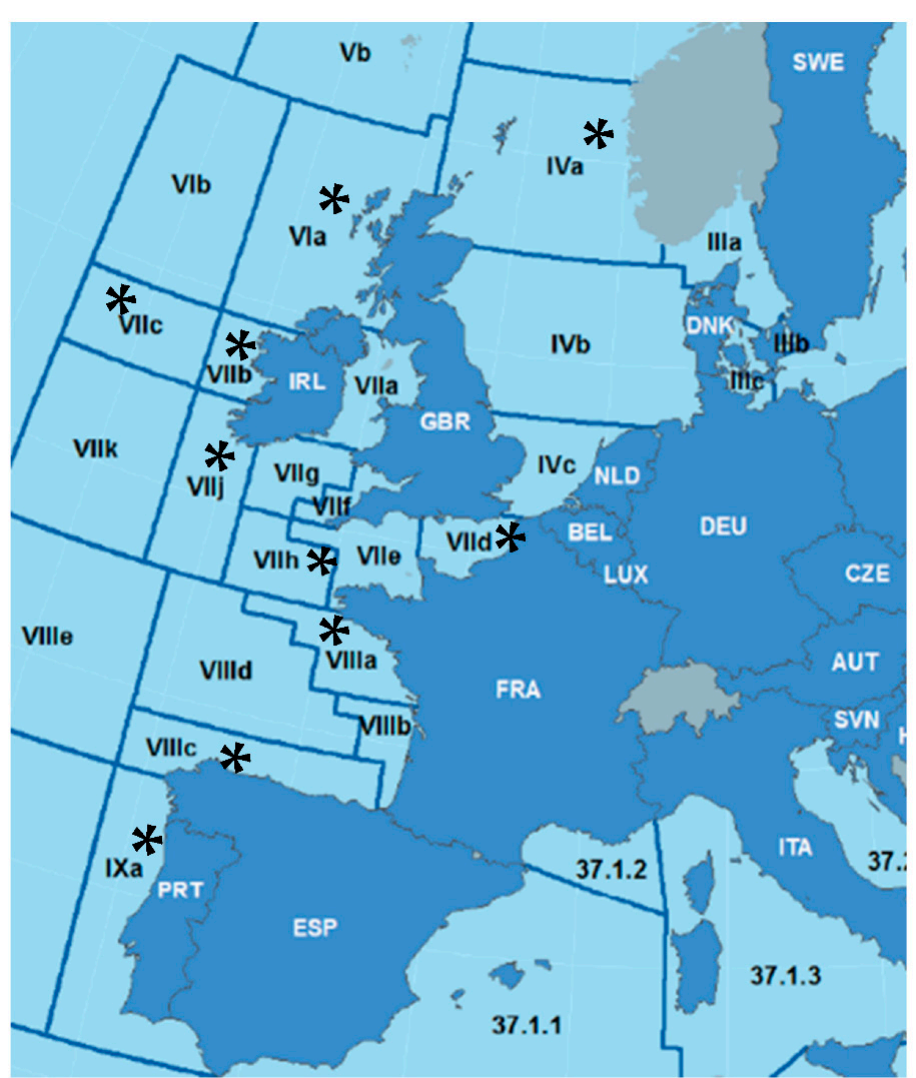
Sampling areas (*) according to ICES Division Areas: IVa (Northern North Sea); VIa (Northwest Coast of Scotland and North Ireland); VIIb (West of Ireland); VIIc (Porcupine Bank); VIIh (Celtic Sea/South); VIIj (Southwest of Ireland/East); VIIIa (Bay of Biscay/North); VIIIc (Bay of Biscay/South); VIIId (Bay of Biscay/ Offshore); and IXa (Portuguese Waters/East).

A total of 873 traceable high-quality samples of *Anisakis* spp. third-stage larvae from *Merluccius merluccius* were donated by the Technical Unit of the Marine Biobank at the Institute of Marine Science (UTB-IIM-CSIC, Vigo). These samples consisted of *Anisakis* specimens originally collected from ten ICES divisions belonging to five subareas (Figure 1): IVa (Northern North Sea); VIa (Northwest Coast of Scotland and North Ireland); VIIb (West of Ireland); VIIc (Porcupine Bank,); VIIh (Celtic Sea/South); VIIj (Southwest of Ireland/East); VIIIa (Bay of Biscay/North); VIIIc (Bay of Biscay/South); VIIId (Bay of Biscay/Offshore); and IXa (Portuguese Waters / East). As a rule, over 75 *Anisakis* juveniles, L3 larvae, were randomly selected from five hakes, from each division sampled from 2018 to 2019 (15 parasites per host), including specimens from the muscular sections: belly flaps (BF) (*N* = 409), loins (LO) (*N* = 252) and tail (TA) (*N* = 72). Additionally, 140 visceral *Anisakis* larvae (VIS) were chosen from five hakes (over 10 parasites per host) caught in subareas VIIj, VIIIc and IXa in the period 2013–2014. 

### 2.2. Taxonomic Identification of *Anisakis* spp. L3 Larvae

Genomic DNA of each larva was obtained employing the commercial kit Wizard Genomic DNA Purification Kit (Promega), according to the manufacturer’s protocol. DNA quality and quantity was checked in a spectrophotometer Nanodrop® ND-2000 (Thermo Scientific, Waltham, MA, USA). Genetic identification of *Anisakis* spp. was carried out using ITS rDNA region as molecular marker. PCR assays of all *Anisakis* larvae were performed in the robotic workstation in total volume of 25 μL containing 1 μL of genomic DNA, PCR buffer at 1× concentration, 0.2 mM nucleotides (Thermo Scientific), 0.3 μM of each NC5/NC2 primers [21] and 0.025 U μL − 1 Dream Taq DNA polymerase (Thermo Scientific). A negative control (no DNA) was used in each PCR assay. The PCR assays were carried out in a T gradient thermocycler (Biometra), under the following reaction parameters: 95 °C for 5 min, 35 cycles at a melting temperature of 95 °C for 30 s, an annealing temperature of 55 °C for 45 s, an extension temperature of 72 °C for 1 min, followed by a final extension period of 72 °C for 7 min. The completed reactions were resolved using 2% agarose gel electrophoresis, Red Safe stained and visualized in Gel Doc™ XR System (Bio Rad, Hercules, California, USA). PCR products were cleaned for sequencing using ExoSap-It (Thermo Fisher Scientific, Massachusetts, USA) for 15 min at 37 °C, followed by inactivation for 15 min at 80 °C. Sequencing was performed by the company STABVIDA (Portugal), and the chromatograms were analyzed using ChromasPro v.1.41 (Technelysium Pty Ltd., South Brisbane, Australia). Two diagnostic nucleotide sites of ITS1 region, 278 and 294, were identified in order to differentiate *A. simplex* (T nucleotide in both sites), *A. pegreffii* (C in both of them) and heterozygotes (two overlapping C/T peaks in both positions) [8]. All generated sequences were also assessed for similarity against known sequences using BLAST (Basic Local Alignment Search Tool) of the National Center for Biotechnology Information (NCBI, Bethesda, MD, USA).

### 2.3. Genetic Diversity and Haplotype Analysis

Genomic DNA of all identified *A. simplex* was amplified at the mitochondrial cytochrome oxidase 2 gene (mtDNA *cox*2), using 211F/210R pair of primers described by Nadler and Hudspeth [22]. PCR reactions were performed as described above and under the following reaction parameters: 95 °C for 5 min, 35 cycles at a melting temperature of 95 °C for 30 s, an annealing temperature of 48 °C for 45 s, an extension temperature of 72 °C for 1 min, followed by a final extension period of 72 °C for 7 min. PCR products were also sequenced and analyzed as described above. 

Multiple alignments of the sequences achieved were constructed using MEGA 7 [23] and analyzed in the software DnaSP v6 [24] in order to know the genetic diversity of *A. simplex* populations. Three sets of mtDNA *cox*2 sequences were defined in DnaSP v6 as three case studies: (1) *A. simplex* sequences grouped by the 10 fishing divisions sampled; (2) *A. simplex* sequences grouped by the 3 muscular fish sections; (3) *A. simplex* sequences of muscular and viscera specimens from divisions VIIj, VIIIc and IXa. The number of haplotypes (N_h_), the haplotype diversity (H_d_), nucleotide diversity (P_i_), number of segregating sites (S) and the average number of nucleotide differences (K) were calculated by DnaSP v6 for all defined sets. Median-joining haplotype networks [25] were constructed using PopART (http://popart.otago.ac.nz (accessed on 13 December 2022)). Neutrality test, Tajima’s D [26] and Fu’s Fs [27] were performed in Arlequin v3.5.2. software [28] with 1000 simulations to analyze the randomness of the DNA sequence evolution by the verification of the null hypothesis of selective neutrality (expected with population expansion). In addition, the genetic structure of *A. simplex* populations was also evaluated by a hierarchical analysis of molecular variance (AMOVA). Pairwise comparisons of F_st_ [29] values between populations were calculated with 1000 permutations. 

## 3. Results

### 3.1. Genetic Identification of *Anisakis* spp. L3 Larvae

A fragment of 905 bp of ITS1-5.8S-ITS2 region from 733 *Anisakis* larvae collected from fish muscular sections and 140 *Anisakis* from fish viscera were amplified and successfully sequenced. According to the diagnostic positions, a total of 695 muscular specimens showed the homozygote pattern of *A. simplex* (T nucleotide in 278 and 294 sites), 12 specimens showed the profile of *A. pegreffii* (C in both positions) and 26 *Anisakis* had a heterozygote genotype (T/C in 278/294 sites). The genetic identity of *Anisakis* specimens from viscera by sequencing of ITS region showed the *A. simplex* pattern in 76 cases, those of *A. pegreffii* in 43 specimens and heterozygote profile in 21 larvae. 

*Distribution of Anisakis species in ICES divisions. A. simplex* was the predominant species in hakes from all divisions sampled, represented the 94.82% of all specimens identified, followed by hybrids *A. simplex* x *A. pegreffii* (3.55%) and *A. pegreffii* (1.64%). *A. simplex* and hybrids were identified in the ten divisions whereas three divisions showed to be sympatric areas for *A. simplex*, *A. pegreffii* and hybrids: IXa (63 *A. simplex*, 7 *A. pegreffii* and 7 hybrids); VIIIa (63 *A. simplex*, 3 *A. pegreffii* and 5 hybrids) and VIIIc (71 *A. simplex*, 2 *A. pegreffii* and 3 hybrids) (Table 1).

Anisakis *species in fish sections.* The predominant species *A. simplex* in European waters represented similar percentages 95.84% in belly flaps, 93.65 % in loins and 93.06% in tails; *A. pegreffii* showed lower percentages in BF (1.47%), LO (1.59%) and TA (2.78%), similar to those for hybrids in BF (2.69%), LO (4.76%) and TA (4.16%) (Table 1). *Anisakis* larvae recovered from viscera from divisions VIIj, VIIIc and IXa showed different results (Table 2). Parasites from the sympatric area IXa for *A. simplex*, *A. pegreffii* and hybrids showed that the predominant species was *A. pegreffii*, doubled to *A. simplex*, whereas *A. simplex* and *A. pegreffii* showed similar values in the VIIIc division. Thus, 53.33% of parasites of viscera from division IXa were *A. pegreffii*, the 22.22% *A. simplex* and 24.44% hybrids for both species. The identification of *Anisakis* species from VIIIc revealed that 43.14% was *A. simplex*; 37.25% was *A. pegreffii;* and 19.61% was hybrids. The results obtained for division VIIj showed the 100% of *Anisakis* identified were *A. simplex.*

### 3.2. Genetic Diversity and Population Structure of *A. simplex*

#### 3.2.1. Case Study 1: *A. simplex* from Different ICES Divisions

A total of 510 mtDNA cox2 sequences were obtained from *A. simplex* collected in hakes from ten ICES divisions. The alignment of all *A. simplex* sequences (483 bp) contained 115 variable sites (S), which resulted in 215 haplotypes. Genetic diversity indices for all divisions are shown in Table 3. The overall value of haplotype diversity (Hd) was 0.938, of nucleotide diversity (Pi) was 0.00723, and the value of the average number of nucleotide differences (K) was 3.49440. The genetic diversity indices calculated for *A. simplex* from each division showed a similar haplotype diversity for all of them ranged between 0.848 for division VIa to 0.970 for VIIIa and with pi values ranged between 0.00496 from VIa to 0.00948 from VIIc. Neutrality test, Tajima’s D and Fu’s, showed negative values statistically significant (*p*-value ˂ 0.05 and *p*-value ˂ 0.02, respectively) for all divisions, except for VIIh whose Tajima’s D value was not significant (*P* = 0.12800), rejecting the null hypothesis, i.e. the population evolves according to the infinite-site model and all mutations are selectively neutral (Table 3). 

**Figure 2:**
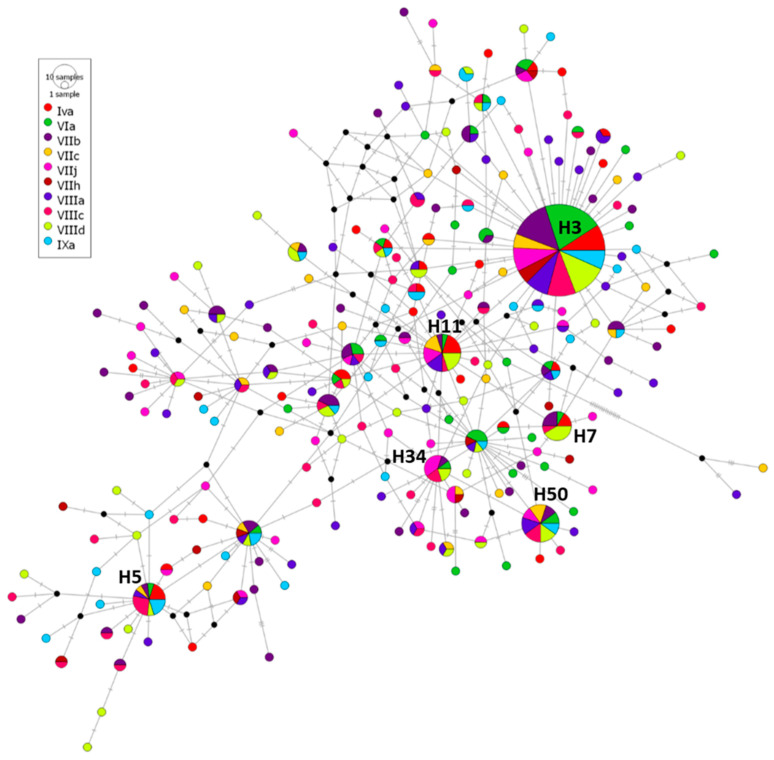
Median-joining haplotype network of *A. simplex* mtDNA cox2 sequences obtained from European hake from 10 ICES division areas. Circles’ size represents the frequency of each haplotype. Hatch marks show the number of mutations distinguishing the haplotypes. Majority haplotypes (including more than 10 sequences) are reported. Black points indicate missing haplotypes.

Median-joining haplotype network of the 510 mtDNA cox2 sequences of *A. simplex* from the 10 divisions studied (Figure 2) was represented, showing the 215 haplotypes obtained by DNAsp. The haplotype H3 was clear majority and the only haplotype shared for all fishing divisions, including 120 sequences (23.53% of the total sequences). It showed relative frequency ranging from 0.169 (division VIIIa) to 0.391 (division VIa). The other more representative haplotypes (those including more than 10 sequences and with maximum relative frequencies of 0.0857) were common only among some of divisions: H50 (20 sequences/3.92%) was common for all locations, except for IVa and VIIh; H11 (20 sequences/3.92%) was shared for all divisions except VIIh and IXa; H5 (15 sequences/2.94%) was in all of them, except in VIIj and VIIh; H7 (12 sequences/2.35%) was common to divisions IVa, VIa, VIIb, VIIIc, VIIId and IXa; and H34 (10 sequences/1.96%) was shared for VIa, VIIb, VIIj, VIIIc, VIIId. 

A total of 168 haplotypes (78.15% of the total 215 haplotypes) were unique for some of the 10 divisions analyzed and, in all cases, were represented for only one sequence, representing more than 44.12% of total haplotypes for each division (Table 3, Figure 2). The population structure of *A. simplex* from the different fishing divisions was explored with AMOVA, showing that 99.75% of genetic variation was explained by differences within populations and 0.25% of genetic variance by differences among populations. The fixation index F_st_ for *A. simplex* among the 10 divisions was 0.00255 (*P* = 0.11241). F_st_ values (pairwise genetic differentiation) among *A. simplex* sequences of the 10 divisions sampled are shown in Table 4. The highest F_st_ values appeared between Via, and the other nine divisions compared with it, ranging between 0.01503 and 0.06270; all of them were significantly different (*p* ˂ 0.05). In contrast, the differences between the other nine populations of *A. simplex* were not statistically significant, and their F_st_ values were lower than those of VIa.

#### 3.2.2. Case Study 2: *A. simplex* from Different Fish Muscular Sections

A total of 510 mtDNA cox2 sequences from *A. simplex* described above were grouped in belly flap (BF), loin (LO) and tail (TA), depending on where it was taken from. The genetic diversity indices calculated for *A. simplex* from each muscular section were similar between them and to those obtained to the 10 fishing divisions (Table 5). The Hd value for BF was 0.942, for LO 0.932 and for TA 0.943, with Pi values of 0.00696, 0.00751 and 0.00783, respectively. Neutrality test, Tajima’s D and Fu’s also showed negative values statistically significant (*p* ˂ 0.05 and *p* ˂ 0.02, respectively) for the three sections. 

**Figure 3:**
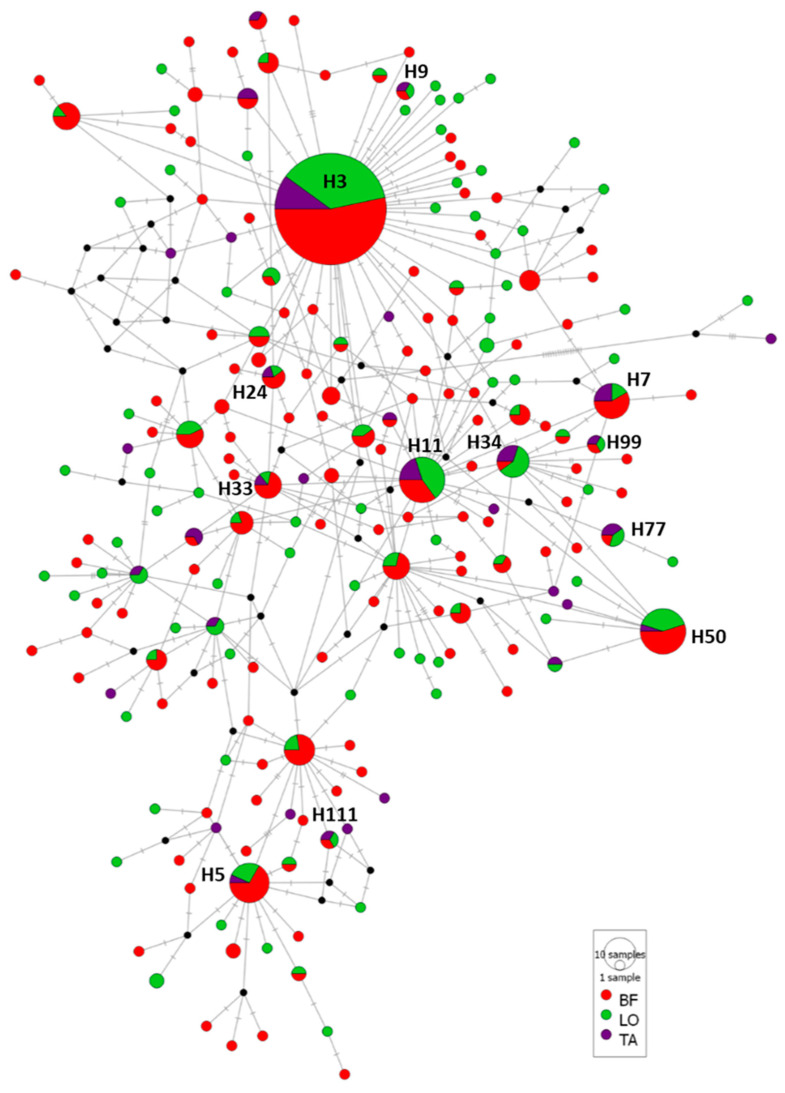
Median-joining haplotype network of *A. simplex* mtDNA cox2 sequences obtained of muscular sections from European hake: belly flaps (BF), loins (LO) and tails (TA). Circles’ size represents the frequency of each haplotype. Hatch marks show the number of mutations distinguishing the haplotypes. Haplotypes shared for the three sections are reported. Black points indicate missing haplotypes.

Median-joining haplotype network of the 510 mtDNA cox2 sequences of *A. simplex* from the three muscular sections was represented (Figure 3). Twelve haplotypes were shared for BF, LO and TA: H3, H50, H11, H5, H7, H34, H33, H24, H77, H9, H99 and H111, being the H3 haplotype, majority in all fishing subareas, the most frequent (64 sequences in BF, with a relative frequency of 0.229), 44 in LO (0.250) and 22 in TA (0.222). Other shared haplotypes showed lower relative frequencies, being higher in haplotypes: H50 was represented in BF with 11 sequences (relative frequency of 0.0393), in LO with 8 sequences (0.0455) and in TA with 1 sequence (0.0185); H11 occurred in BF with 7 sequences (0.025), in LO with 9 sequences (0.0511) and in TA with 4 (0.0741). The network revealed 19 haplotypes were shared between BF and LO, 4 between BF and TA and only 3 were common to LO and TA, with relative frequencies ranging from a maximum of 0.025 and a minimum of 0.00357. 

A total of 177 haplotypes (82.33% of the total 215 haplotypes) were unique for some of the three muscular sections analyzed. Remarkably, 100 unique haplotypes were found in BF, represented by between one and four sequences of each of them; 63 unique haplotypes in LO (including 1 or 2 sequences per haplotype) and 14 exclusive haplotypes in TA (with 1 sequence per haplotype) (Table 5, Figure 3). The AMOVA results showed that 100% of genetic variation was explained by differences within three populations (BF, LO and TA) since slightly negative values of variance were obtained among groups. The fixation index F_st_ for *A. simplex* sequences among the three sections was also negative (−0.00056) (*P* = 0.58065). F_st_ values among *A. simplex* sequences of the three divisions sampled showed negative low values when BF/LO (−0.00042) and LO/TA (−0.00247) and a low positive value for the comparison BF/TA (0.00020), but in all of the cases, the values were statistically non-significant.

#### 3.2.3. Case Study 3: *A. simplex* from Fish Muscle Versus Viscera

A total of 201 mtDNA cox2 sequences from *A. simplex* were compared, 156 corresponding to those of muscular sections of hakes from divisions IXa, VIIj and VIIIc (obtained in above subsection) and 45 to viscera of the hakes from the same divisions. The alignment of all *A. simplex* sequences (483 bp) contained 78 variable sites and 116 haplotypes. Genetic diversity indices for two groups were Hd = 0.961, Pi = 0.00773 and K = 3.73567, and they were similar between sequences of specimens from muscle and viscera. Thus, Hd values were 0.958 for muscle group and 0.971 from viscera, and Pi values of 0.00752 and 0.00846, respectively. Neutrality test, Tajima’s D and Fu’s, also showed negative values statistically significant (*p*-value ˂ 0.05 and *p*-value ˂ 0.02, respectively) for the two groups (Table 6).

**Figure 4:**
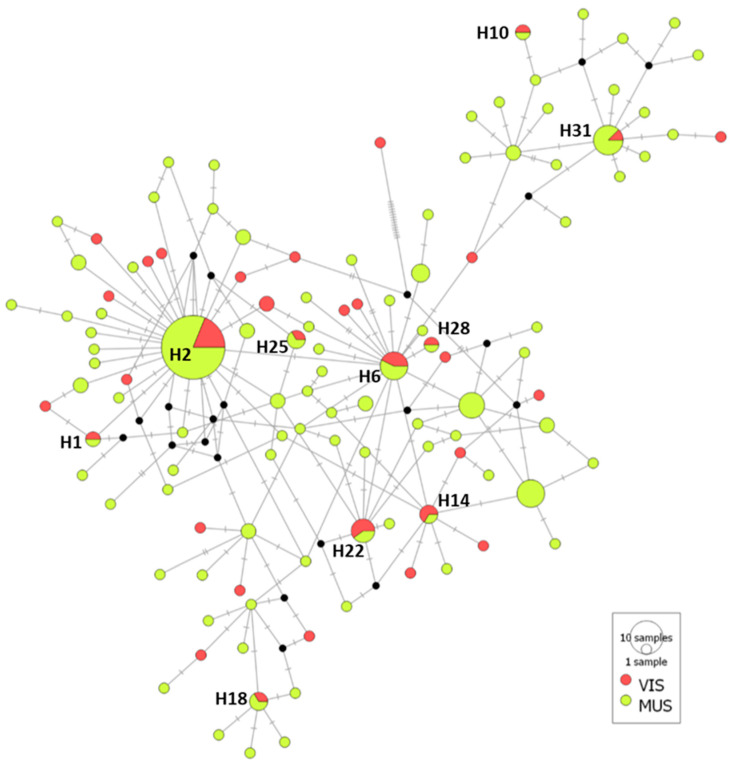
Median-joining haplotype network of *A. simplex* mtDNA cox2 sequences obtained from viscera (VI) and muscle (MUS) of European hake. Circles’ size represents the frequency of each haplotype. Hatch marks show the number of mutations distinguishing the haplotypes. Haplotypes shared for VIS and MUS are reported. Black points indicate missing haplotypes.

Median-joining haplotype network of the 201 mtDNA cox2 sequences of *A. simplex* from muscle and viscera showed the 116 haplotypes obtained; 10 of them (8.64% of the total haplotype) were shared between both groups (H2, H31, H6, H22, H14, H18, H25, H1, H10, H28). Among them, the haplotype name H2 (equivalent to H3 of the comparative of fishing divisions) was the more representative with 37 sequences (18.41% of total sequences) and with relative frequency values of 0.156 for *A. simplex* of viscera and 0.192 for muscle group (Figure 4). A total of 104 haplotypes (89.66% of the total 116 haplotypes) were unique for viscera or muscle: 23 haplotypes were exclusively of viscera, representing a 69.70% of total viscera haplotypes (22.12% of total haplotypes); 81 haplotypes were unique of muscle, 87.10% of total muscular haplotypes (77.88% of total haplotypes). In both groups, the haplotypes included only one or two sequences (Table 6/Figure 4). The AMOVA results showed that 99.87% of genetic variation was explained by differences within two populations, muscular and viscera, and 0.13% was explained by differences between them, with a statistically non-significant F_st_ of 0.00126 (*P* = 0.05415).

## 4. Discussion

### 4.1. Genetic Identification of *Anisakis* Species of European Hake

The genetic differentiation between the sibling species of *A. simplex* complex and hybrids *A. simplex* x *A. pegreffii* is crucial to understand their epidemiology, ecology and zoonotic potential. Several molecular markers have been described for this purpose, among them PCR-RFLPs profiles of ITS rDNA region [8]; nuclear markers sequencing of EF1 α1 nDNA region [30] or beta-tubulin gene [31] and an ARMS-PCR protocol based on nas 10 nDNA [32]; mitocondrial markers as cytochrome c oxidase subunit II (cox2) [33] or even panels of microsatellite loci to distinguish between *A. simplex* and *A. pegreffii* [34,35]. However, the results in detecting hybrids are not always consistent. In this study, the genetic identification between *A. simplex*, *A. pegreffii* and their hybrids was performed used ITS1 marker [8]. Some authors indicate this marker can overestimate *Anisakis* hybrid genotypes [15,30], but others consider it useful in detecting F1 hybrids and later generation back-crosses [31,36]. Moreover, Steinauer et al. [36] suggest that ITS1 is more sensible than other molecular markers to detect hybrids due to its high copy number. 

To carry out the study of population structure of *A. simplex*, we used those larvae that showed an unequivocal homozygote pattern of *A. simplex* (T nucleotide in 278 and 294 sites) in ITS1 region, according to Abollo et al. [8]. The number of *A. pegreffii* and hybrids obtained in this study were scarce to perform a robust genetic population study. Thus, mtDNA cox2 gene for all the identified *A. simplex* was also sequenced since it is a common marker used for studies of population structure of the genus *Anisakis* [34,37,38,39] and of numerous species of parasites [40,41,42,43], due to their high substitution rate and maternal inheritance. 

The molecular identification of *Anisakis* larvae infecting the muscle of *M. merluccius* showed clearly that *A.* simplex is the predominant species in the 10 ICES divisions. This represents the 94.82% of all specimens identified, which is in accordance with previous studies [13,44]. *A. pegreffii* constituted only the 1.64% of the total specimens identified, and it was found exclusively in Portuguese waters (IXa) and Bay of Biscay (VIIIa and VIIIc, respectively). Noticeably, hybrids *A. simplex* x *A. pegreffii* also appeared mainly in these fishing divisions, confirming that the Spanish and Portuguese coast are sympatric areas for both species and their hybrids [3,8,10,13,14,44]. Likewise, the comparative study of *Anisakis* species from belly flaps, loins and tails showed similar results since more than 93% of identified species were assigned to *A. simplex* in the three fish sections, whereas a lower number of *A. pegreffii* and hybrids were found (ranging from 1.47 to 4.76% of total specimens). However, when *Anisakis* specimens of viscera from the sympatric areas (IXa, VIIIc) were analyzed, *A. pegreffii* doubled those values obtained for *A. simplex*. Likewise, a higher number of hybrids was also detected in both divisions. These results are completely different from those obtained for muscular sections from the same fishing divisions. Cipriani et al. [10] described the distribution of *Anisakis* species in viscera and muscle of European hake from IXa division and reported that the relative distribution of *A. simplex* doubled to *A. pegreffii* in viscera, but this proportion increased in flesh, being the prevalence almost five times higher for *A. simplex* than for *A. pegreffii*. Pascual et al. [3] obtained similar results when they identified *Anisakis* species from viscera of hake from the Iberian Peninsula (*A. simplex* 68.2%, A. *pegreffii* 30.3%, hybrids 1.5%). Our results reinforce the values obtained in previous studies. Several studies have highlighted the tissue specificity of different *Anisakis* species, suggesting that *A. pegreffii* has a lower penetration index in the fish muscle than *A. simplex* [45,46,47] and that *A. physeteris* is not capable of migrating in the flesh of the fish [48]. 

### 4.2. Genetic Diversity and Population Structure of *A. simplex* Infecting *M. merluccius* from Different ICES Divisions

The genetic diversity indices calculated for mtDNA cox 2 sequences of *A. simplex* from the different fishing divisions showed a high haplotype diversity but low nucleotide diversity for all populations (except for VIa) whose Hd, Pi and K value were lower than for the other populations. These values are translated at network in a high number of total haplotypes (215), with a high percentage of unique haplotypes (78.14%) with very low relative frequency. A single haplotype (H3) was clearly majority and distributed in all divisions. It indicates a clear connection of the *A. simplex* from Northern North Sea to Portuguese coast. Moreover, the distribution of this haplotype seems even wider since it corresponds to the same unique haplotype (H5) for *A. simplex* usually noted all around the North Atlantic from *Sebastes mentella* (East Greenland, Tampen- Northern North Sea, Barents Sea Bear Island; [38]), and with the most frequent haplotype (H1) of *A. simplex* from *Clupea harengus* (Norwegian Sea, North Sea, English Channel and Baltic Sea; [39]). The sequence of H3 haplotype found in this study also matches with the sequence deposited in GenBank for the highly migratory fish *Mola* from the Mediterranean Sea [49] and with those obtained from the definitive hosts *Delphinus delphis* from Galicia [50] and *Stenella coeruleoalba* from Adriatic Sea [51]. The high frequency of this haplotype and its wide geographic and host range could indicate that it represents the most ancestral haplotype of this species [39]. 

Neutrality tests showed high negative values statistically significant, rejecting the null hypothesis (i.e., the population evolves according to the infinite-site model and all mutations are selectively neutral, point to an excess of rare polymorphisms due to recent demographic expansion events and/or positive selection). These results are similar to those recorded in populations of *A. simplex* collected in *C. harengus* [39] and in populations of *A. simplex* from *S. mentella*, both from North East Atlantic fishing grounds [38]. The genetic diversity obtained for *A. simplex* is also consistent with those described for *A. simplex* parasitizing the Pacific sardine (*Sardinops sagax*) from the California Current system [37]. 

The molecular variance analysis (AMOVA) revealed a high intra-population genetic variation (99.75%) whereas a low percentage (0.25%) was explained for geographical differences among fishing divisions. This is in agreement with the results observed for *A. simplex* infecting *C. harengus* from North East Atlantic [39]. Population pairwise Fst values showed a slight level of differentiation, statistically significant, when the population of VIa division was compared with the other nine populations, suggesting a certain level of genetic sub-structuring in the population of the Northwest coast of Scotland. Fst values for the remaining populations showed a lack of genetic structuring, meaning there is not geographic separation among *A. simplex* from different fishing divisions. 

High genetic diversity and lack of genetic structure have also been found in other anisakid species, such as *Contracaecum rudolphii* A, parasitizing great cormorants (*Phalacrocorax carbo*) sampled in different localities from Sardinia [42]. A high gene flow is often described in parasitic nematode populations from aquatic vertebrates, especially fish, resulting in a slight genetic structure of parasite populations [52]. Two main factors have been noted to invigorate this gene flow between parasite metapopulation: (1) complex life cycles: high host range and low host specificity in highly migratory host populations and (2) high infection intensities in definitive hosts, because of multiple (and even heavily) infected intermediate and paratenic hosts [3,11,42,53]. 

Despite the very limited population structure, present results suggest a certain level of genetic sub-structuring in *Anisakis* population of the Northwest coast of Scotland (VIa). In European waters, a similar slight geographical separation has already been described with *A. simplex* from *C. harengus* and *Balenoptera acutorostrata* from Norwegian Sea [11,39]. Three factors have been suggested as responsible: (1) the Norwegian *C. harengus* stock as an important food source for *B. acutorostrata*; (2) the migration pattern of *B. acutorostrata*, mainly distributed from the Atlantic coast of France to Greenland; and (3) the lower proportion of some species of the Delphinidae in Norwegian Sea, comparing with other European waters. Up to now, the genetic structure of *Anisakis* species in the Northwest coast of Scotland, including Hebrides Islands, had not been studied. A different hake stock in that VIa division could be related with the slight genetic differentiation of *A. simplex*. At present, ICES assumes the existence of two stocks of the European hake within the Northeast Atlantic Ocean: the northern stock (ICES Division IIIa, Sub-areas II, IV, VI and VII and Divisions VIIIa, b, d) and the southern stock (ICES Divisions 8c and 9) [1,2]. However, studies based on otolith chemistry and microsatellites did not support this population structure [54,55,56]. Recently, using High-throughput sequencing, it was determined that European hakes from the Norwegian Sea were genetically different from those of East Bay of Biscay (both belonging to the northern stock) and from those at the northwestern Iberian Peninsula (southern stock), whereas these latter two locations matched genetically [57]. Further studies are needed to clarify population structure of the European hake, and they should include not-studied areas, such as the North Sea, the Celtic Sea and the Northwest Coasts of Scotland and Ireland. On the other hand, although cetaceans are highly migratory species, they often show subpopulations with high fidelity to small areas. Thus, for example, a population of killer whales *Orcinus orca* from Hebrides Islands showed a different pigmentation pattern when compared with neighboring populations [58]. Cheney et al. [59] also suggested the existence of three parapatric communities of bottlenose dolphins (*Tursiops truncatus*) in Scottish coastal waters, two of them located in the west coast. Evidences of the occurrence of hybrids between bottlenose dolphins and Risso’s dolphins (*Grampus griseus*) from Hebrides have also been observed [60]. Moreover, Inner and Outer Hebrides have important colonies of grey seal *Halichoerus grypus,* since UK has the 40% of the world’s population and 95% of all grey seal in Europe [61]. These colonies of grey seal, more sedentary than other cetacean species, could also be implicated in that slight genetic differentiation of *A. simplex* in division VIa. 

### 4.3. Genetic Diversity and Population Structure of *A. simplex* from Fish Sections

For the first time, the genetic diversity and population structure of *A. simplex* from different fish sections of European hake was evaluated. The results showed that the genetic diversity indices for mtDNA cox 2 sequences of *A. simplex* of BF, LO and TA were similar for the three muscular sections and also similar to those sequences of *A. simplex* from viscera. Likewise, neutrality test and the population genetic variation among parasites obtained from the four different fish sections pointed out the lack of genetic structure among them. The same haplotype H3, majority in all fish divisions, is also clearly the most prevalent and it is interchangeably both in the three muscular sections and in viscera (named H2) with similar relative frequencies. The high frequency of this haplotype in the 10 fishing divisions and in the 4 fish sections shows that it is well adapted to different micro-and macroenvironmental conditions. A high number of unique haplotypes were also found for the four fish sections. A plausible explanation for the high number of unique haplotypes found for the four fish sections might be that these haplotypes play an important role for adaptation or colonization of different microenviroments. For this reason, the infection dynamic and pathogenicity of the haplotypes identified, both those predominant and minority, should be further analyzed in order to establish their hypothetical adaptative strategies related to tissue specificity. 

## 5. Conclusions

This study provides novel insights into the distribution of *Anisakis* spp. parasitizing the European hake along the ICES fishing areas and in the different fish sections. Remarkably, *A. simplex* is the predominant species in the edible parts of *M. merluccius* in all divisions studied. However, in sympatric fishing areas for *Anisakis* (VIIIc and IXa), the number of *A. pegreffii* in viscera was similar to *A. simplex*, or even greater.

Our results revealed that *A. simplex* constitutes a single genetic population along the 10 fishing divisions studied although a statistically significant genetic sub-structuring was also found for *A. simplex* in the Northwest coast of Scotland, which could indicate the presence of a subpopulation in this area. We found a high haplotype diversity for this parasite species in hake but a clear dominant haplotype in all the divisions and fish sections studied. More experimental and analytical studies are necessary in order to test if the different haplotypes described present any difference in resilience and adaptability, which could confer a greater evolutionary fitness.

## Figures and Tables

**Table 1:** Taxonomic identification of *Anisakis* larvae from muscular sections analyzed by sequencing of ITS rDNA.

ICES Area		As	Ap	Hyb	Total
IVa	BF	62	0	3	65
	LO	8	0	0	8
	TA	1	0	0	1
	Total	71	0	3	74
VIa	BF	57	0	0	57
	LO	13	0	1	14
	TA	1	0	0	1
	Total	71	0	1	72
VIIb	BF	59	0	0	59
	LO	11	0	1	12
	TA	4	0	0	4
	Total	74	0	1	75
VIIc	BF	42	0	0	42
	LO	23	0	1	24
	TA	9	0	1	10
	Total	74	0	2	76
VIIj	BF	24	0	0	24
	LO	31	0	2	33
	TA	6	0	0	6
	Total	61	0	2	63
VIIh	BF	37	0	1	38
	LO	35	0	0	35
	TA	2	0	0	2
	Total	74	0	1	75
VIIIa	BF	2	0	0	2
	LO	49	2	3	54
	TA	12	1	2	15
	Total	63	3	5	71
VIIIc	BF	37	0	0	37
	LO	28	2	3	33
	TA	6	0	0	6
	Total	71	2	3	76
VIIId	BF	11	0	0	11
	LO	37	0	1	38
	TA	25	0	0	25
	Total	73	0	1	74
IXa	BF	61	6	7	74
	LO	1	0	0	1
	TA	1	1	0	2
	Total	63	7	7	77
Overall		695	12	26	733

As: *A. simplex*; Ap: *A. pegreffii*: Hyb: hybrid genotype between *A. simplex* and *A. pegreffii*.

**Table 2:** Taxonomic identification of *Anisakis* larvae from viscera analyzed by sequencing of ITS rDNA.

ICES Area	As	Ap	Hyb	Total
VIIj	44	0	0	44
VIIIc	22	19	10	51
IXa	10	24	11	45
Overall	76	43	21	140

As: *A. simplex*; Ap: *A. pegreffii*; Hyb: hybrid genotype between *A. simplex* and *A. pegreffii*.

**Table 3:** Genetic diversity indices and neutrality test based on mtDNA cox2 sequences of *A. simplex* parasitizing European hakes from ten ICES divisions.

ICES Areas	N	Nh	Nuh	Pi	Hd ± SD	K	S	Tajima’s D		Fu’s Fs	
								D	P	Fs	P
IVa	44	27	13	0.00613	0.930 ± 0.030	2.96300	30	−1.93422	0.00800 *	−24.42734	0.00000 *
VIa	64	34	15	0.00496	0.848 ± 0.045	2.39633	30	−2.00076	0.00700 *	−26.84892	0.00000 *
VIIb	69	43	22	0.00718	0.937 ± 0.024	3.46974	41	−1.93982	0.00800 *	−26.18191	0.00000 *
VIIc	35	26	14	0.00948	0.965 ± 0.020	4.57815	44	−2.06327	0.00200 *	−19.69666	0.00000 *
VIIj	50	33	19	0.00740	0.953 ± 0.021	3.57633	34	−1.77822	0.01000 *	−26.07247	0.00000 *
VIIh	18	13	6	0.00755	0.902 ± 0.066	3.64706	17	−1.00685	0.12800	−6.41729	0.00100 *
VIIIa	59	45	25	0.00860	0.970 ± 0.015	4.15546	57	−2.25027	0.00100 *	−25.85918	0.00000 *
VIIIc	60	42	19	0.00752	0.956 ± 0.019	3.63277	36	−1.74418	0.01700 *	−26.07312	0.00000 *
VIIId	65	38	18	0.00675	0.938 ± 0.022	3.25962	32	−1.66792	0.02500 *	−26.27973	0.00000 *
IXa	46	33	17	0.00764	0.966 ± 0.018	3.69082	32	−1.67002	0.02700 *	−26.01534	0.00000 *
Overall	510	215	-	0.00723	0.938 ± 0.009	3.49440	115	−2.31355	0.00000 *	−25.42466	0.00100 *

Number of sequences analyzed (N), number of haplotypes (Nh), number of unique haplotypes (Nuh), nucleotide diversity (Pi), haplotype diversity (Hd) with their relative standard deviation (SD), average number of nucleotide differences (K), number of variable sites (S), Tajima’s D (D) and Fu’s F (Fs) statistics with their *P*-values (D, significance level 0.05 and Fs, significance level 0.02). * Significant values.

**Table 4:** Population pairwise F_st_ values from mtDNA cox2 sequences among *A. simplex* from 10 fishing divisions. * significant value (significance level 0.05).

	IVa	VIa	VIIb	VIIc	VIIj	VIIh	VIIIa	VIIIc	VIIId
IVa	-								
VIa	0.02373 *	-							
VIIb	−0.00631	0.02802 *	-						
VIIc	0.00118	0.02149 *	−0.00203	-					
VIIj	0.01078	0.04431 *	0.00325	−0.00585	-				
VIIh	0.00614	0.06270 *	0.00116	−0.00112	0.01541	-			
VIIIa	−0.00411	0.01503 *	−0.00565	−0.01071	−0.00473	0.00953	-		
VIIIc	0.00025	0.04427 *	0.00058	0.00612	−0.00073	0.00587	−0.00036	-	
VIIId	−0.00296	0.02871 *	−0.00479	−0.00732	−0.00082	0.01133	−0.00553	0.00177	-
IXa	−0.00303	0.04545 *	−0.00188	0.00673	0.01137	−0.01415	0.00173	−0.00629	0.00031

**Table 5:** Genetic diversity indices and neutrality test based on mtDNA cox2 sequences of *A. simplex* of European hakes from muscular sections: belly flaps (BF), loins (LO) and tails (TA).

	N	Nh	Nuh	Pi	Hd ± SD	K	S	Tajima’s D		Fu’s Fs	
								D	P	Fs	P
BF	280	135	100	0.00696	0.942 ± 0.011	3.36193	81	−2.21675	0.00000 *	−25.84468	0.00000 *
LO	176	97	63	0.00751	0.932 ± 0.016	3.62675	74	−2.21567	0.00100 *	−25.90587	0.00000 *
TA	54	33	14	0.00783	0.943 ± 0.023	3.78337	45	−2.09405	0.00400 *	−25.94050	0.00000 *
Overall	510	215	-	0.00723	0.938 ± 0.009	3.49440	115	−2.31355	0.00000 *	−25.42466	0.00100 *

Number of sequences analyzed (N), number of haplotypes (Nh), number of unique haplotypes (Nuh), nucleotide diversity (Pi), haplotype diversity (Hd) with their relative standard deviation (SD), average number of nucleotide differences (K), number of variable sites (S), Tajima’s D (D) and Fu’s F (Fs) statistics with their *p*-values (D, significance level 0.05 and Fs, significance level 0.02). * significant values.

**Table 6:** Genetic diversity indices and neutrality test based on mtDNA cox2 sequences of *A. simplex* from viscera (VIS) and muscle (MUS) of European hakes.

	N	Nh	Nuh	Pi	Hd ± SD	k	S	Tajima’s D		Fu’s Fs	
								D	P	Fs	P
VIS	45	33	23	0.00846	0.971 ± 0.016	4.08687	45	−2.09847	0.00500 *	−25.91039	0.00000 *
MUS	156	93	81	0.00752	0.958 ± 0.012	3.63383	61	−2.04939	0.00200 *	−25.84799	0.00000 *
Overall	201	116	-	0.00773	0.961 ± 0.010	3.73567	78	−2.19753	0.00000 *	−25.76160	0.00000 *

Number of sequences analyzed (N), number of haplotypes (Nh), number of unique haplotypes (Nuh), nucleotide diversity (Pi), haplotype diversity (Hd) with their relative standard deviation (SD), average number of nucleotide differences (K), number of variable sites (S), Tajima’s D (D) and Fu’s F (Fs) statistics with their *P*-values (D, significance level 0.05 and Fs, significance level 0.02). * significant values.

## Data Availability

Not applicable.
